# Tourism research from its inception to present day: Subject area, geography, and gender distributions

**DOI:** 10.1371/journal.pone.0206820

**Published:** 2018-11-02

**Authors:** Andrei P. Kirilenko, Svetlana Stepchenkova

**Affiliations:** The Department of Tourism, Recreation and Sport Management, University of Florida, Gainesville, Florida, United States of America; Institut Català de Paleoecologia Humana i Evolució Social (IPHES), SPAIN

## Abstract

This paper uses text data mining to identify long-term developments in tourism academic research from the perspectives of thematic focus, geography, and gender of tourism authorship. Abstracts of papers published in the period of 1970–2017 in high-ranking tourist journals were extracted from the Scopus database and served as data source for the analysis. Fourteen subject areas were identified using the Latent Dirichlet Allocation (LDA) text mining approach. LDA integrated with GIS information allowed to obtain geography distribution and trends of scholarly output, while probabilistic methods of gender identification based on social network data mining were used to track gender dynamics with sufficient confidence. The findings indicate that, while all 14 topics have been prominent from the inception of tourism studies to the present day, the geography of scholarship has notably expanded and the share of female authorship has increased through time and currently almost equals that of male authorship.

## 1. Introduction

Recent years have evidenced an increased interest to tourism as a knowledge system [1] and to bibliometric analysis of tourism research output [2–6]. Systematic evaluation of scientific output in a particular field of study using bibliometrics (statistical analysis of publications) is usually conducted from one of three main perspectives: an individual author, an academic journal, and an academic field [7]. At the individual author level, authorship is examined in terms of academic leadership, productivity, and collaborative networks, using indicators such as the number of publications (e.g., [8]), impact (mainly through citation analysis, e.g., [9]), co-citations (e.g., [2]), and co-authorship statistics [5,10]. At the academic journal level, studies are primarily concerned with issues of knowledge dissemination and transfer as well as journal quality and impacts [11–13]. Such studies are often used as guidelines to evaluate the quality of research output in academic institutions, make funding decisions, and help institutions formulate recommendations for tenure and promotion.

From the macro-level viewpoint of the academic field itself, which is the focus of this study, systematic examination of published scholarship is used to track evolution of the discipline, identify new trends and developments, point to gaps in knowledge and areas of inconsistency in research findings, suggest directions for future research, and, more generally, provide an up-to-date overview of the field [6,14]. For such a wide-ranging and diverse discipline as tourism, which is infused with contributions from various fields of inquiry, the analysis of its structural properties is of a particular value. Such analyses can outline not only current relationship dynamics of tourism with the ‘traditional’ study areas like sociology or marketing but also with more closely connected areas such as hospitality or leisure studies. For example, research by Cheng et al. [15] revealed that scholarly tourism journals have been diverging from leisure and well-being domain from which tourism research originated.

Evaluation of scholarly contributions in a particular field of study has social significance as well [7,15]. Two issues of social importance, in particular, have attracted attention from tourism researchers: geography and gender. Strong interest to these issues from the community of tourism scholars is manifested in conferences’ academic agendas, calls for papers for special journal issues, and recurring debates in professional networks such as TRINET. With respect to the geography issue, a long-standing concern has been the existing dominance of the Western perspective in tourism research and the underrepresentation of views not encompassed by the Western philosophical, social, and political tradition [16,17]. While geography typically represented by the first author’s institution cannot be equated with study’s perspective, viewpoint, or philosophy, the growing diversity in geography of tourism scholarship could serve as a feasible proxy.

Similarly, issues of gender parity in research, journal editorship, and education and administration have been brought into focus [18,19]. A recent issue of *Anatolia* journal, which is entirely devoted to the topic of gender in tourism academy, argues that “[g]iven that gender is so central to our identity formation and the structures of our societies, we question how it can be received as peripheral to the dominant discussions of the evolution of tourism knowledge and research production” [19]. In the articles tracking the scholastic achievement, the gender-related findings are typically reduced to the statements of women’s underrepresentation in the ‘most productive scholars’ lists (e.g., [20]). The direct tracing of the dynamics of gender representation in tourism scholarship has not yet been conducted. This is not surprising considering the variety of names from various corners of the world that are present in scholarly output and, until recently, the lack of methods to track the gender attribution of these names with high degree of confidence.

Recent developments in natural language processing and text mining allow analyses of voluminous data corpora that were not possible before. A document like an academic article deals with several issues at once and, thus, belongs to several subject areas, albeit with different ‘strength’ [21,22]. However, determining topical ‘strength’ has been a persistent problem in content analysis until very recently. The unsupervised classifiers such as Latent Dirichlet Allocation (LDA) or Principal Component Analysis (PCA) and supervised classifiers such as Support Vector Machine (SVM), or Naïve Bayes deliver classification of documents into multiple categories, with category weights numerically expressed. Categories and their weights are discerned based on clusters of words that repeatedly co-occurred in textual segments, providing a more measured and objective classification. Further, with development of probabilistic methods of gender identification based on social network data mining and availability of online gender name-databases, the issue of gender identification in academic scholarship can be tackled with high degree of confidence. While text data mining methods have been percolating into tourism research (e.g., [23,24]), study utilizing text mining approaches for content analysis of unstructured data are still in single digits [25–27]. Thus, recent methodological developments paved the way to the analyses conducted in this study to determine as objectively as possible subject areas of tourism research and their evolution over more than 40 years, as well as geography and gender distribution of tourism scholarship.

## 2. Tourism scholarship: Literature review

In bibliographic studies, the ancillary information that accompanies each journal publication (e.g., year of publishing or number of citations) allows quick summaries, aggregation, and production of trends. The textual information, however, contained in the articles or their abstracts is more difficult to summarize and interpret. Studies that are concerned with subject areas of tourism research and/or developments in the field method-wise lean to one of the two main approaches: content analysis or quantitative relational analysis. Content analysis and its multiple variants include categorization of textual units using pre-specified or inductively derived lists of disciplinary foci, topical areas, keywords, or headwords, producing frequency counts with subsequent tabulation. The relational approach is an assemblage of quantitative techniques (e.g., co-citation analysis, network analysis) that compute similarity scores between units of analysis (e.g., articles or their authors) with subsequent clustering of those units and then visualize solutions with drafted networks of related articles/citations, scholars, and fields of study [6,10,28]. So far, the content analysis approach has been more popular with tourist researchers; however, relational techniques are gaining grounds with latest development in computational methods (see Table 1).

**Table 1:** Identification of subject areas in tourism research.

Authors	Study years	Journals analyzed*	Data	Subject areas	Method of topic identification**	Geography	Gender
[31]	1973–1998	ATR	subject index, headwords	multiple headwords as indicators of topical areas	headword analysis	countries by author	-
[14]	1973–2003	ATR	subject index, headwords	27 subject areas	content analysis of the subject indices	continents and international regions by author, no trends	-
[32]	1994–2004	ATR, CIT, IJTA, JRR, JTR, JTS, JVM, TA, TCC, TE, TG, TM	2868 articles	21 topic areas	content analysis based on random sampling from the article pool	-	-
[28]	1994–2007	ATR, TM	334 articles	keywords as indicators of topics	content analysis; relational analysis	-	-
[15]	1970–2011	59 tourism-related journals	journal mission statements	29 disciplinary focuses	content analysis	-	-
[30]	2000–2009	tourism: ATR, JTR, TM; hospitality: JHTR, IJHM, CHQ	2834 articles	20 subject areas	previous studies, expert opinions	research rankings by country of author, no trends	-
[6]	2008–2014	ATR, CIT, IJTR, JTR, JTTM, JST, SJHT, TE, TG, TM	2545 articles	12 research topics and 41 sub-topics	co-citation analysis, cluster analysis, text mining	contribution to topics by country of author, no trends	-
[29]	2000–2014	THR	292 articles	19 research themes	pre-identified themes	-	-
[26]	1975–2017	ATR	858 abstracts	dynamics of separate words as indicator of research interest	text mining: LDA, SVM	-	-
This study	1973–2017	ATR, JTR, TM	6110 abstracts	14 subject areas	text mining: LDA	GIS visualization of temporal dynamics of scholarship by country of author	probabilistic approach of gender identification

* ATR—Annals of Tourism Research; CHQ—Cornell Hospitality Quarterly; CIT—Current Issues in Tourism; IJHM—International Journal of Hospitality Management; IJTA—International Journal of Tourism Analysis; IJTR—International Journal of Tourism Research; JHTR—Journal of Hospitality & Tourism Research; JRR—Journal of Recreation Research; JST—Journal of Sustainable Tourism; JTR—Journal of Travel Research; JTS—Journal of Tourism Studies; JTTM—Journal of Travel & Tourism Marketing; JVM—Journal of Vacation Marketing; SJHT—Scandinavian Journal of Hospitality and Tourism; TA -Tourism Analysis; TCC—Tourism Culture and Communication; TE—Tourism Economics; TG—Tourism Geographies; THR—Tourism and Hospitality Research; TM—Tourism Management.

** SVM—Support Vector Machine; LDA—Latent Dirichlet Allocation

### 2.1. Content analysis studies

An example of content analysis with pre-identified or inductively inferred categories would be Strandberg et al. [29] who evaluated scholarship published in journal of *Tourism and Hospitality Research* using 18 study areas provided by the journal itself as its scientific scope: “hospitality and tourism operations, marketing and consumer behavior, HR management, eTourism/eTravel. technology, planning and development, policy, performance and financial management, strategic implications, environmental aspects, forecasting and prediction, revenue management, impact assessment and mitigation, globalization, research methodologies, leisure and culture, risk management, and change management” (p. 9). The researchers added one more category, education, and coded 292 collected papers by their primary themes. Using three sequential 5-year periods, the authors were able to follow the dynamics of the primary themes in the journal scholarship.

Park et al. [30] identified 20 subject areas of tourism research published in six major tourism and hospitality journals (2,834 articles in total), drawing on previous studies and expert opinions. These areas included “attraction management; crisis and safety management; destination marketing and management; tourism development and residence perception; economic impact and econometrics; education; geographical issues; general marketing; image and branding; information technology; meetings, incentives, conventions, and exhibitions, including festivals and fairs; tourism planning; politics, policy, legal, and governmental issues; supply chain management; market segmentation; special interests tourism; service management; sustainable tourism and eco-tourism; tourists’ perceptions and behavior; and other” (p. 384). Classification articles into these pre-established categories was conducted by checking their title, abstract, keywords, and, in some of the cases, the entire content of the paper.

Ballantyne et al.[32] examined 2,868 academic articles published in 12 major tourism journals from the list provided by McKercher et al. [12] for a 20-year period (1994–2004), categorizing them into 21 topical areas. The areas were inductively derived based on examination of randomly drawn 200 articles from the total pool: tourist/visitor studies; destinations; tourism planning; marketing; cultural tourism; economic issues; tourism impacts; tourism trends; tourism research issues & methods; hospitality; eco-tourism; sustainable development; special events; transport; management; human resource management; environmental interpretation; tourism policy; tourism education and training; business tourism; and sports and leisure (p. 150). ‘Manual’ approach to content analysis required the researchers to select a stratified random subsample of 144 articles in order to provide a more detailed analysis of research trends in top four subject areas and scholarship in top three journals.

An example of content analysis using ‘proxy’ data is demonstrated by Cheng et al. [15] who identified the disciplinary foci of 59 tourism-related journals and tracked changes over three time periods. Researchers evaluated 21 disciplinary foci provided in the Goeldner et al. [33] list (e.g., anthropology, psychology, sociology, economics, marketing, etc.), found the list insufficient, and added eight supplementary disciplinary focuses: cultural/heritage study; management and administration; finance; computer science/technology; gerontology; literature; medicine, and philosophy/religion. Then, journal mission statements were examined to identify the disciplinary areas on which each particular journal was focusing. At this step, researchers followed the protocol of content analysis and calculated the inter-rater agreement. The final counts were used to illustrate growth of tourism-related disciplinary foci over time.

### 2.2. Relational techniques

As can be seen from the discussed examples, the content analysis approach and its results are dependent on the researcher’s individual perspective in selecting categories for coding. Further, since the coding involves human raters, the more complex the system of categories is, the more difficult it is to maintain the efficiency of the analysis and the adequate inter-rater reliability. Journal articles are multidimensional textual units, but raters must force them into a specific topical category, introducing another source of bias based on raters’ preferences. One of the ways to escape the problem with uni-dimensionality would be to use an article’s ‘tagging words’ provided by the authors themselves (keywords) or by the journal (headwords). Thus, Swain et al. [31] identified subject areas of papers published in *Annals of Tourism Research* based on headwords taken from the journal’s subject index. Top ten out of 1,830 headwords were: impacts; organizations; development; research & development; United States; Third World; tourism; international tourism; planning; transport; tourism, study of; hotel; and conferences [31]. The words were interpreted as indicative of topical research interests.

Similarly, Xiao and Smith [14] used the *Annals’* subject index to discern the knowledge domains in the journal papers. Fifty-two headwords were identified that represented eight subject areas which citation frequencies were rising: “typology of tourists, community and development, alternative experience/product, sociocultural aspects and change, geopolitical regions/focus, literature/research/methods, marketing and management, and environment” (p. 496). Twenty-seven headwords were grouped into nine categories of decreasing popularity: “economics, industry and transportation, hospitality, recreation, impacts, North America, tourism (in a conventional/narrow sense), Third World, and sociology” (p. 496). The authors, however, acknowledged that the keyword and headword analyses provided inconsistent results, highlighting the subjectivity of the selected tagging words, as well as a large amount of subjective reasoning involved in aggregating the tagging words into larger themes or categories [31].

The information that is common to any two articles, whether it is keywords, headwords, authors, or references, allows for producing measures of similarity that can serve as a foundation for quantitative relational techniques. For example, co-citation analysis is based on the idea that the more cited works the two particular articles share, the closer these two articles are conceptually [28]. Yuan et al. [6] employed a modification of co-citation analysis, which they named bibliographic coupling: “Quantitatively speaking, the more common references two papers cite, the more closely the two papers relate to each other and the higher its BC strength” (p. 5). The articles were clustered based on the similarity scores but cluster labeling, that is, identification of subject areas, involved reading titles and abstracts of articles in the individual clusters. To facilitate spotting topics for each cluster, text data mining approach was employed [34]; it generated the top five terms for each cluster for further labeling by two tourism field experts.

With more relevance to the goal of this particular paper, Mazanec [26] tested whether LDA and SVM text data mining methods can detect change in the language of tourism research in order to answer a broad question of “whether, over the decades, the study of tourism has changed focus and touched on new issues or has been largely reiterating traditional viewpoints” [26]. The study found statistically significant temporal differences in frequencies of identified word groups; however, the study did not interpret these word groups in terms of research topics, or subject areas and advocated pursuing the scientific issue of text mining further to detect the reasons and contents of change. With this in mind, the study identified three specific research questions for investigation: (1) What are the subject areas in tourism research from its inception in the early 1970s to the present day and their temporal dynamics? (2) Where did tourism research develop and what is the spatial dynamics of its geography? (3) What is the gender of tourism scholarship and its temporal distribution? The study aims at objective and reliable identification of spatiotemporal distributions of tourism subject areas, geography, and gender using published tourism scholarship as the primary data source. The study selected LDA as a text mining method, which is described in section 3.2.

## 3. Method

### 3.1 Data collection

We collected abstracts from the “Big Three” [35] tourism journals: *Annals of Tourism Research* (*ATR*), *Journal of Travel Research* (*JTR*), and *Tourism Management* (together with its predecessor, the *International Journal of Tourism Management*) (*TM*) for a period of more than 40 years. These three foremost journals in the tourism field have maintained their leading positions for a long time, as confirmed by their journal rankings (www.scimagojr.com), impact factors, citation indices, and published literature reviews [5,7,12,13,35,36]. They can be viewed as analogous to ‘prestige press’ newspapers in political, media, and communication studies that act as trendsetters in the field of tourism research [8,10,14,26,31,37,38]. Abstracts rather than whole documents were selected as the most precise and concise representation of articles’ essence, including its subject area.

All the abstracts stored in the Scopus publication database for these three journals were downloaded, resulting in 8,890 article abstracts with publication dates ranging from 1974 until August 2017. Note that the publication date may differ from the date an article becomes available to readers. Standard procedures of data quality control were then applied (e.g., [39]). First, changing data formatting issues were resolved. Then, abstracts from other journals accidentally included in the database and duplicate entries were removed, resulting in 7,427 articles. Of these entries, 6,110 papers included abstracts (*ATR*: 1,676 abstracts; *JTR*: 1,413 abstracts; and *TM*: 3,021 abstracts). The entries with missing abstracts mainly represented editorials, reviews, and similar publications; also note that the practice of requiring the abstracts was not yet firmly established at the beginning of the search period. Those entries without abstracts were used in the geographical and gender trend analysis, but not in the subject area content analysis.

**Fig 1:**
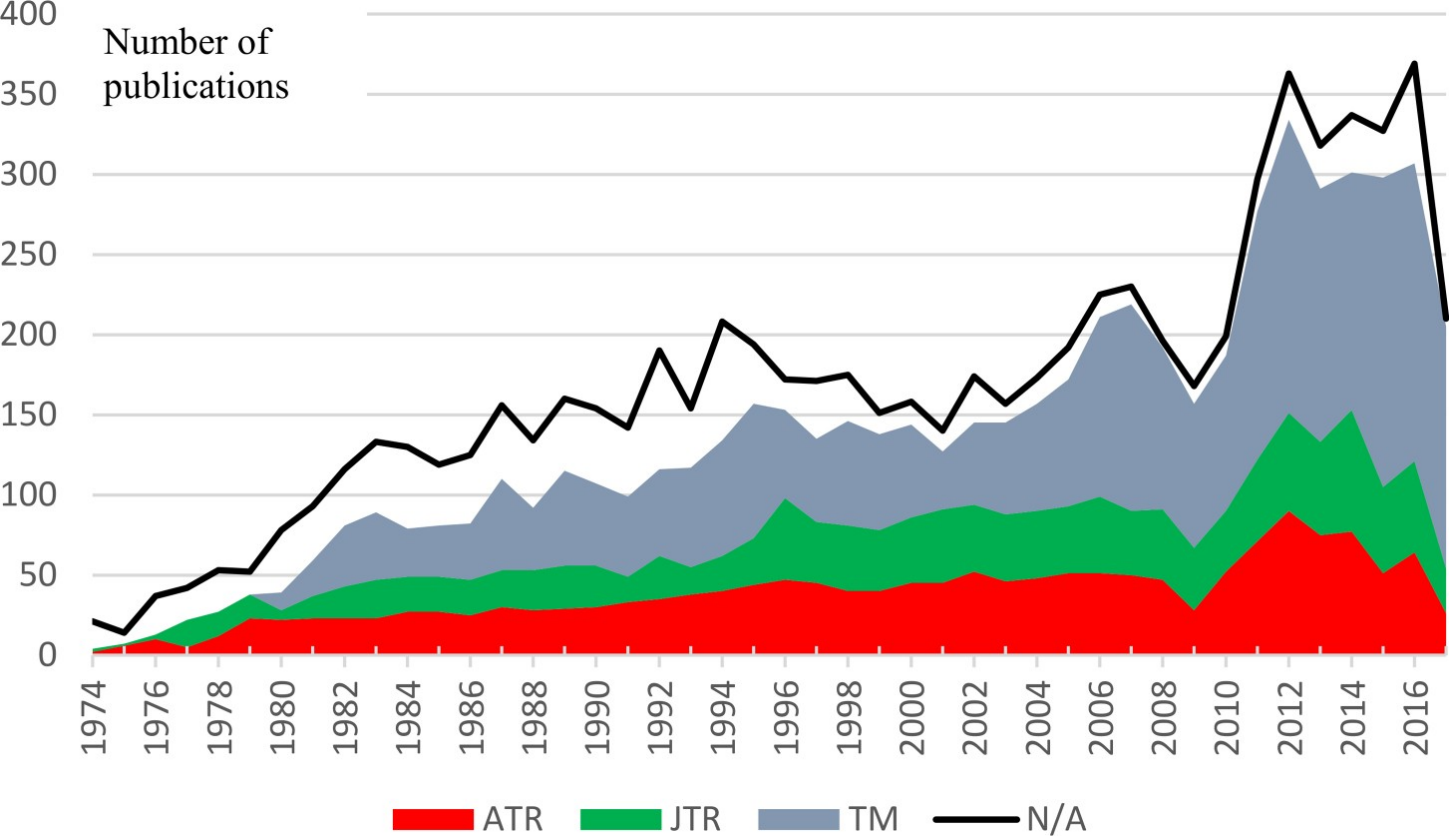
Distribution of collected abstracts in the Annals of Tourism Research (ATR), Journal of Travel Research (JTR), and Tourism Management (TM) over time (article/annum). Note that the reduced number of article in 2009 is not a data collection artifact: compare Tourism Management volume 28 (2007, 1592 pages), volume 30 (2009, 936 pages) and 32 (2011, 1496 pages). The solid line represents the total number of journal papers in Scopus database with and without the abstracts.

Collected publication metadata contained the following information: publication ID in Scopus database; ISSN; title; date, volume, issue, and number of pages of the publication; name and affiliation of the first author; and the number of papers citing the publication. The author affiliation field was missing for 6.3% of entries, the author’s name was missing in 0.9%, and all other fields were missing in less than 0.1% of entries. Fig 1 shows the temporal distribution of collected data. Only the first author’s name was present in Scopus database; thus, all 7,427 papers, including those with missing abstracts, were then processed through Microsoft Academic Search to extract the records for the remaining authors. This search identified 7,045 papers; 382 papers were not present in the Microsoft publication database.

### 3.2 Content analysis with LDA

The Latent Dirichlet Allocation method (LDA) [40] models a collection of documents where each document contains multiple topics (latent variables) represented through its words (observed variables). The LDA approach then attempts to find latent topics based on the distribution of the observed words over the documents. The LDA model was successfully used to extract content from the abstracts of papers published in the Proceedings of the National Academies of Science [41]. Talley et al. [42] used a similar method to extract the topics from ca. 80,000 grant proposals that received awards form the US National Institutes of Health (NIH). Sugimoto et al. [43] applied LDA to the titles and abstracts of doctoral dissertations defended in library and information science with the goal of extracting dominant topics and identifying changes in the field over time. In the applied sciences, Moro et al. [44] analyzed the full texts of business intelligence publications with LDA and identified research trends and prospective research topics in the field. Sing et al. [45] used LDA to process over 25,000 abstracts from medical journals to identify research topics related to spinal care. Zhang et al. [46] used LDA to extract the topics from the abstracts of medical and biomedical papers published by 20 leading UK universities with the overall goal of estimating the “newsworthiness” of research in respective areas for the general mass media.

Extracting the subject areas of publications from the collected abstracts and examining their spatial and temporal variability was achieved by performing an automated search for similar patterns of words appearing in different documents. Formally, we constructed a probabilistic model of the abstracts collection through a Bayesian analysis of their texts. The analysis included the following steps, as outlined in [47]. Steps 1–8 were performed with the RapidMiner data mining platform [48], while Step 9 was performed with a program written in Python using an open-source Python LDA package (http://pythonhosted.org/lda):

1. Tokenization: breaking the sentences into discrete words and word combinations;
2. Part-of-speech (POS) tagging: marking each word in the sentence according to the corresponding part of speech;
3. Removal of stop words: elimination of common words (such as “the”) in the English language that are irrelevant in identifying the specific themes appearing in the texts. The stop words include prepositions, articles, pro-nouns and other frequent words that are equally likely to be present in documents from different topics. We used the Porter stop word list supplied with RapidMiner platform and then a custom stop list (S2 File);
4. POS-based text reduction: elimination of all words other than those tagged as “noun” or “adjective”. Different types of automated text analysis concentrate on words from different POSs (e.g., adjectives are useful for sentiment analysis); however, topical analysis is primarily based on the texts’ nouns. It has been shown (e.g., [49]) that eliminating all other POS words improves article topic extraction. We found, however, that the inclusion of adjectives (e.g., “historical”, “authentic”, etc.) improves topic recognition in tourism related texts;
5. Stemming: reducing inflected words to their word roots, performed using the Porter stemmer [50];
6. Bigram collection: joining sequential tokens. Bigrams allow an analysis based on a group of words as opposed to a single word. For example, a documents containing words “tourism industry” would produce two unigram tokens: "tourism" and "industry" and one bigram: “tourism_industry”;
7. Synonym replacement: merging words with identical meanings such as “tourism industry" and "tourist industry". See S2 File for the synonym list;
8. Co-occurrence matrix creation: First, all words left in the documents are joined into a global dictionary containing N words. Then, each document is represented by an N-dimensional vector based on the presence or absence of a specific word from the dictionary in that document. Then, a co-occurrence matrix is formed by the vectors representing all documents. Three different schema of co-occurrence matrix creation were explored: binary term occurrence, term occurrence—inverse document occurrence (TF/IDF), and term occurrence. The term occurrence schema was selected because it returned the most consistent topics.
9. Topic extraction with LDA. We used the latent Dirichlet allocation using Gibbs sampling Python package (https://github.com/lda-project/lda).
10. Interpretation of words from identified latent topics as tourism concepts belonging to a particular subject area in tourism research.

The issues of selecting the number of topics (K) and values of the model parameters α and β are related to step 9 of LDA application. The formal method of selecting K value based on maximization of a model fit metrics (such as log likelihood) has been criticized in the literature for returning a very large number of topics, many of which are not semantically meaningful (e.g., see the influential paper by a team of researchers from Facebook, University of Maryland, and Princeton University [51]). Instead, it has been suggested that model selection should focus on topic interpretability because “there is no gold-standard list of topics to compare against for every [textual data] corpus” [51]. Subsequently, we processed the abstracts by extracting K latent topics, varying K from 10 to 30, and manually evaluating the extracted topics each time. We found that higher K values tended to return topics with similar content. In contrast, lower values of K tended to return very complex topics. Based on this preliminary analysis and following the recommendations in [51], we selected K value of 14.

Two other model parameters, α and β, define the theoretical distribution of topics over the documents. A literature review showed that the commonly accepted values are α ≈ 0.1 and β = [0.01, 0.1] (e.g., [41]), with a lower α returning fewer dominant topics per document and a lower β returning topics with less similarity. Accordingly, we selected α = 0.1 and β = 0.01. To validate the β selection, we additionally ran the model with the values β = 0.05 and β = 0.1 but found only insignificant changes in the topics expressed as a slightly higher degree of topic intersection.

## 4. Results

### 4.1 Subject areas: What is being published?

The analysis of the abstracts resulted in a 14-topic solution, in which each topic was represented by multiple words with different weights. By consulting the original abstracts in the database for each topical cluster, the interpretive concepts for subject areas were identified. Then, these concepts were joined under one “umbrella” name. To provide an example, the ten most representative words for topic 5 were *service*, *satisfaction*, *quality*, *value*, *relationship*, *attribute*, *custom*, *intention*, *brand*, and *airline* (Table 2). The interpretive concepts were identified as follows: *customer satisfaction; service quality; purchase intention; value;* and *product attributes*. These concepts were joined under the subject area ‘service quality and satisfaction’. To illustrate the result in more detail, we created a supplementary S1 File, which provides representative abstracts from the database that obtained the highest scores on their respective subject areas. The abstract with the highest score for the subject area "service quality and satisfaction" is presented below (1000 is the maximum score).

*“In highly competitive markets*, *customer satisfaction is a key driver of performance*, *making its measurement and management crucial*. *Most studies on customer satisfaction take an aggregate standpoint and do not consider segment-specific differences in attribute importance*. *In this article*, *the authors report on customer satisfaction with alpine ski resorts*. *They hypothesize that personal*, *situational*, *and product factors moderate the relationship between attribute performance and overall satisfaction*. *The results show that these factors indeed influence the attribute-performance-satisfaction relationship*. *Theoretical and managerial implications of these findings are discussed*.*”* (Score: 892; abstract # 42449160073.)

**Table 2:** Fourteen topical clusters extracted from the abstracts of three main academic journals on tourism, 1974–2017. Each topic is represented by multiple terms; only the first ten words with the highest weights are included. Note that the original terms were represented by word roots; the terms were converted to representative nouns and adjectives to improve readability.

ID	Subject Area	Word Cluster
1	Tourism as a social phenomenon	culture, social, place, politics, role, authentic, identity, historical, power, relationship
2	Image and risk	image, tourism destination, risk, behavior, perception, trip, family, response, destination image, media
3	Attractions	visit, attraction, nature, site, management, national, area, park, heritage, value
4	Tourism industry	industry, management, tourism industry, sector, business, organization, hospitality, competition, firm, organizational
5	Service quality and satisfaction	service, satisfaction, quality, value, relationship, attribute, custom, intention, brand, airline
6	Modeling and forecasting	model, method, forecast, application, technique, methodology, measure, series, efficient, system
7	Conferences	hotel, state, event, city, publication, copyright, cost, association, conference, rate
8	Tourist experience and motivation	experience, factor, motivation, response, scale, knowledge, tourist experience, group, dimension, framework
9	Market segmentation	market, segment, tour, operator, strategy, product, agency, tourism market, travel agency, agent
10	Decision making process	international, decision, choice, vacation, pattern, holiday, product, domestic, accommodation, spatial
11	Tourism demand	country, demand, price, expenditure, income, growth, season, period, foreign, show
12	Governing tourism development	economy, region, policy, case, government, problem, resort, island, area, tourism development
13	Sustainable tourism	environment, active, sustainable, interest, participation, leisure, recreation, life, involvement, action
14	Local communities	community, resident, attitude, benefit, local, rural, perception, negative, positive, tourism development

**Fig 2:**
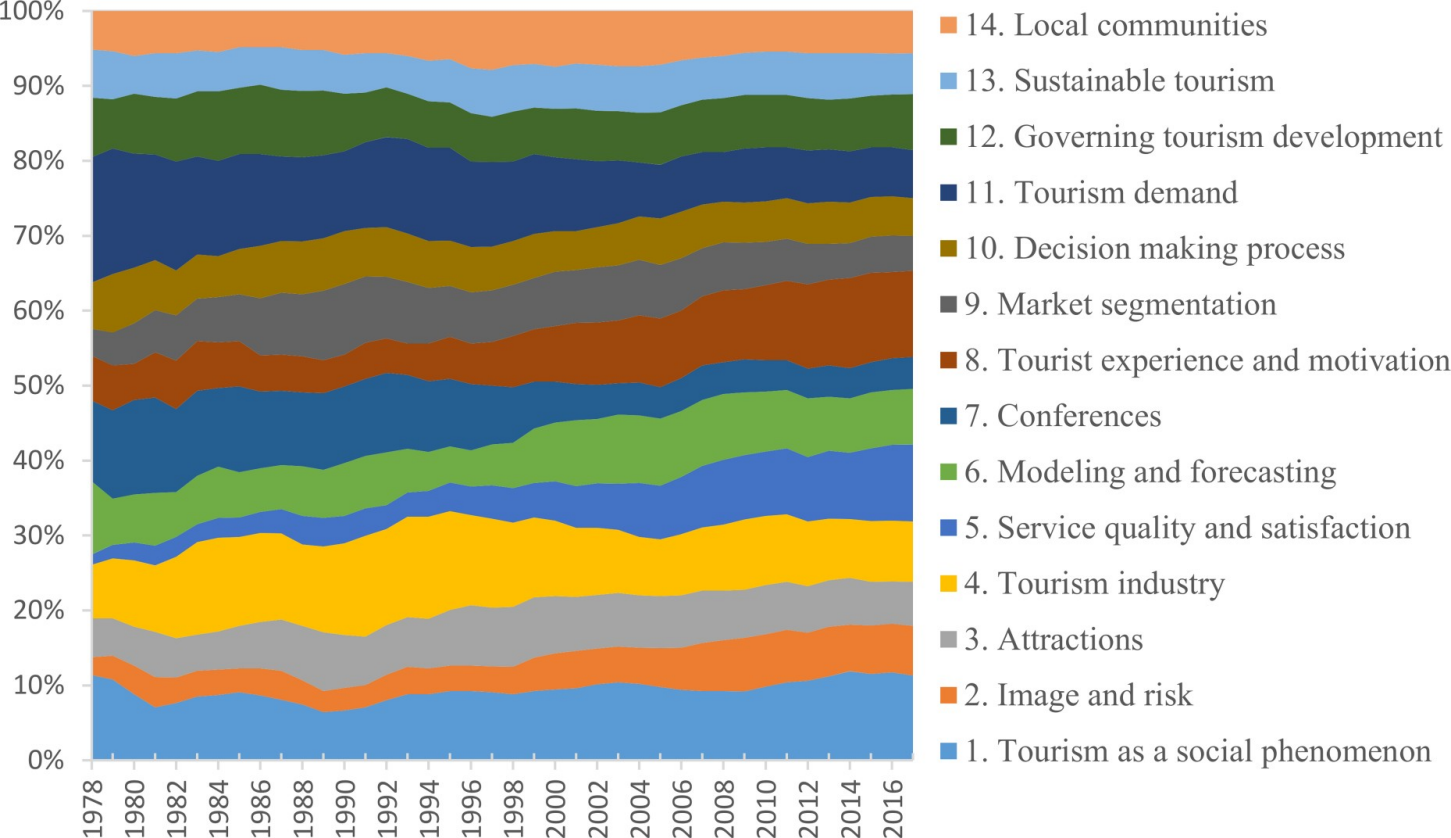
The change in distribution of publication topics over time (5-year running mean).

The temporal dynamics of popularity of any single subject area (as a share of the entire volume) changes across time (Fig 2). For example, subject area ‘tourism demand’ (# 11) falls in popularity, while the popularity of ‘service quality and satisfaction’ (#5) increases. The comparative interest in the issues of sustainable tourism (# 13) and tourism and local communities (# 14) remains relatively constant. Overall, at present, across all three journals, the issues represented by subject areas ‘tourism as a social phenomenon’ (# 1), ‘service quality and satisfaction’ (# 5), and ‘tourist experience and motivation’ (# 8) are slightly more popular compared to the issues emphasized by other areas.

**Fig 3:**
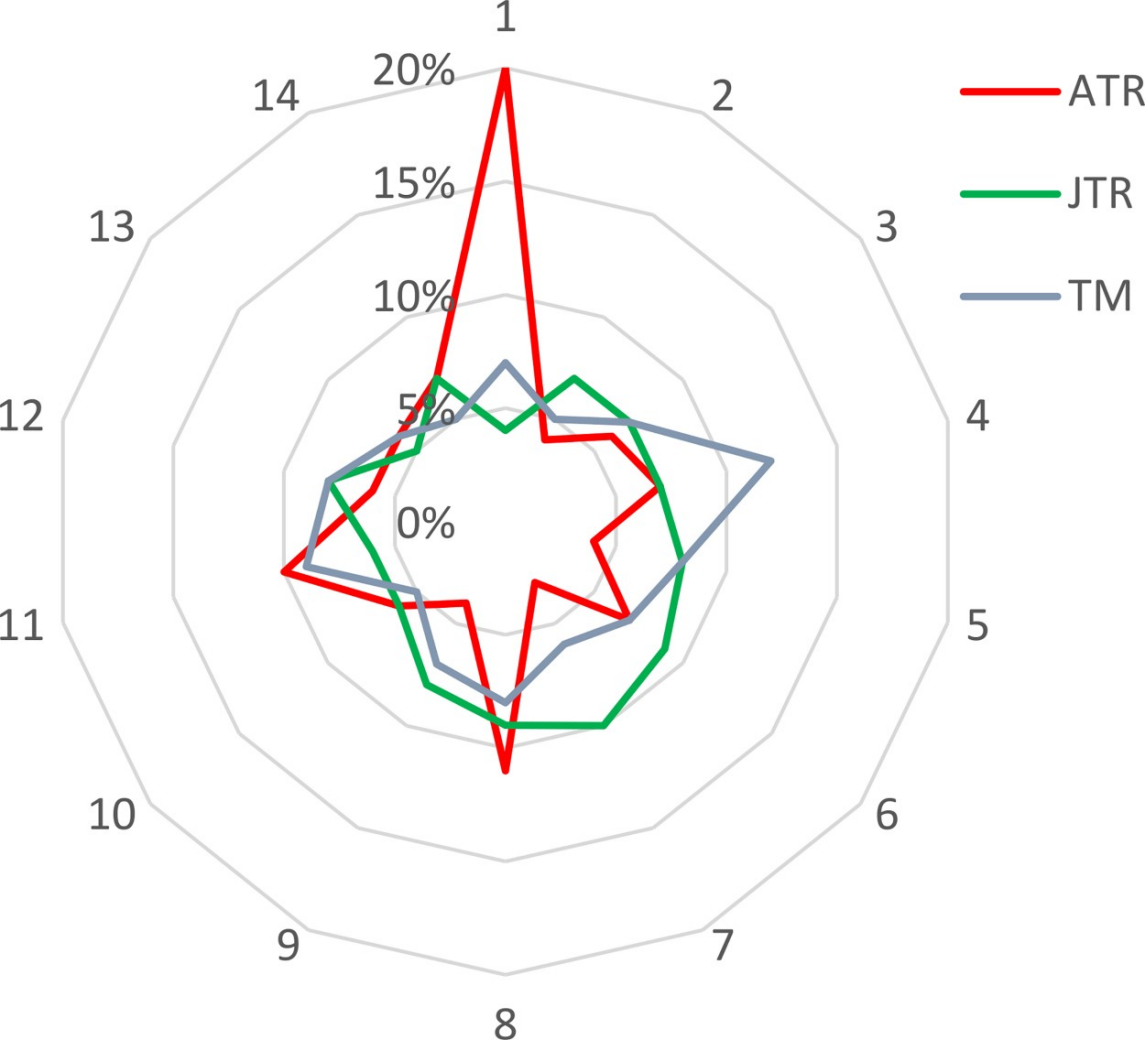
Comparative distribution of subject areas (percentage) over the entire time period of 1972–2017 in *Annals of Tourism Research* (*ATR*), *Journal of Travel Research* (*JTR*) and *Tourism Management* (*TM*). The areas are numbered as follows: 1: Tourism as a social phenomenon; 2: Image and risk; 3: Attractions; 4: Tourism industry; 5: Service quality and satisfaction; 6: Modeling and forecasting; 7: Conferences; 8: Tourist experience and motivation; 9: Market segmentation; 10: Decision making process; 11: Tourism demand; 12: Governing tourism development; 13: Sustainable tourism; 14: Local communities.

The results (Table 3, Fig 3) indicate that some of the identified topics are common to all three journals, while others are more journal-specific. According to LDA analysis, *ATR* is highly interested in studies that view tourism as a social phenomenon and involve a variety of disciplinary perspectives. This is quite consistent with the journal self-identification as a “social sciences journal focusing upon the academic perspectives of tourism. In this role, *ATR* is structured by the research efforts of a multidisciplinary community of scholars” [36]. Subject area of tourism as a social phenomenon, which draws heavily on theoretical developments in anthropology and sociology and heavily involves qualitative methods of analysis, is published disproportionately more often in *ATR*. LDA analysis identified the higher interest of *TM* in the issues related to tourism industry and tourism demand, as compared to the other two publications, consistent with *TM* orientation as an outlet “concerned with the planning and management of travel and tourism” [36]. *JTR* “focuses on travel and tourism behavior, management and development… with diversity in research topics and methodologies” [36]. In analysis, *JTR* emerged as a well-rounded journal topic-wise that demonstrates more interest in modeling and forecasting than do the other two journals and publishes more conference announcements, primarily for the TTRA annual conference.

**Table 3:** Percentage of papers in specific subject areas: Time period and journal.

	ATR				JTR				TM			
Topic	1972–1987	1988–1997	1998–2007	2008–2017*	1972–1987	1988–1997	1998–2007	2008–2017*	1972–1987	1988–1997	1998–2007	2008–2017*
1	17	16	19	25	1	2	4	6	6	7	7	8
2	3	3	4	5	4	6	7	10	2	2	5	7
3	5	7	6	5	6	7	8	5	7	7	8	7
4	9	7	6	7	7	6	7	7	15	19	12	10
5	3	3	4	5	3	4	8	11	3	4	7	11
6	5	8	8	6	10	8	9	9	7	4	8	8
7	4	4	3	2	21	19	6	5	7	8	4	5
8	7	8	9	16	7	5	9	13	4	3	8	10
9	3	5	5	3	8	10	8	7	8	9	8	6
10	6	7	5	6	7	6	6	6	5	6	6	6
11	17	12	9	7	9	7	7	6	15	13	9	7
12	8	7	6	5	7	7	9	8	12	6	6	8
13	7	6	6	7	4	4	5	5	5	6	6	6
14	5	7	8	5	5	8	6	6	4	6	6	5

*2017 data are extrapolated based on the first eight months of the year.

### 4.2 Geography: Where do they publish from?

**Fig 4:**
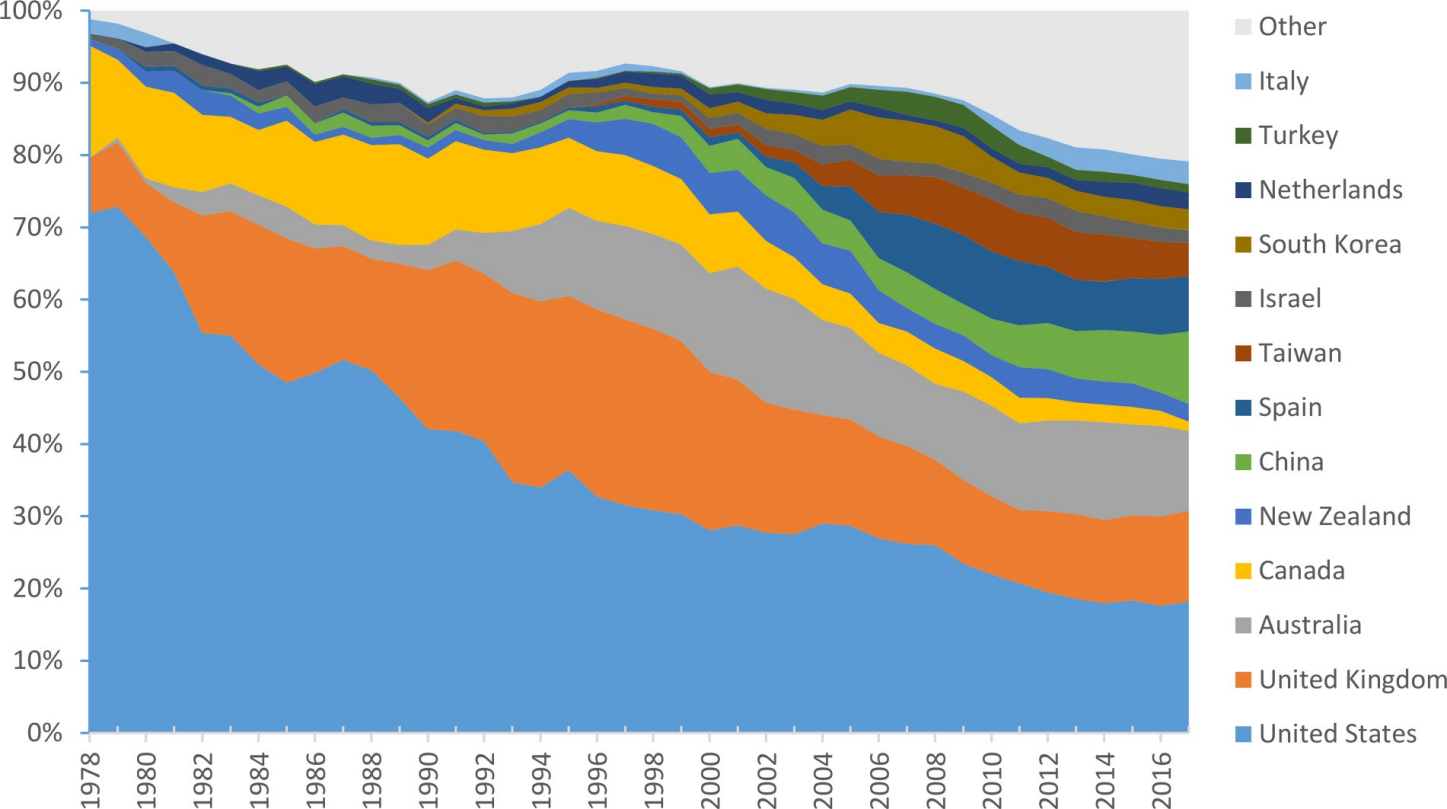
Relative number of publications per country. Publication country is defined from the affiliation of the first author. The countries shown have at least 1% of the total number of publications or at least 3% of publications in any 5-yeat period. To smooth over temporal variability, the figure shows 5-year running mean.

We used the first author’s affiliation data to discern the geographical pattern of tourism research. In total, the tourism journals published papers led by authors from 83 countries (Table 4). Fig 4 shows the change in the relative number of papers published in various countries over time. Note that the figure shows only countries from which at least 1% of the overall paper volume within the study period was published, or at least 3% of the number of papers within any sequential 5-year period. All other countries are merged into the “Other” category. Note also that while the affiliation was missing in only 6.3% of the papers, the distribution of the missing papers over time was not uniform. At the beginning of the research period (up to 1995), the percentage of papers with missing affiliations in each year was high (mean = 18%). However, in 1996 and beyond, the percentage of missing affiliations was sharply reduced (mean = 1%). In Fig 4, the papers with missing affiliations are ignored.

**Table 4:** First author’s country of affiliation.

Country	1972–1987		1988–1997		1998–2007		2008–2017*		Total*	
US	291	(40%)	330	(27%)	414	(26%)	486	(18%)	**1521**	**(25%)**
UK	92	(13%)	232	(19%)	236	(15%)	314	(12%)	**874**	**(14%)**
Australia	17	(2%)	92	(8%)	205	(13%)	304	(11%)	**618**	**(10%)**
Canada	65	(9%)	98	(8%)	85	(5%)	55	(2%)	**303**	**(5%)**
China	7	(1%)	14	(1%)	69	(4%)	225	(8%)	**315**	**(5%)**
Spain	3	(0%)	5	(0%)	84	(5%)	197	(7%)	**289**	**(5%)**
Taiwan	0	(0%)	4	(0%)	61	(4%)	144	(5%)	**209**	**(3%)**
New Zealand	10	(1%)	35	(3%)	70	(4%)	81	(3%)	**196**	**(3%)**
S Korea	0	(0%)	7	(1%)	66	(4%)	75	(3%)	**148**	**(2%)**
Israel	10	(1%)	15	(1%)	32	(2%)	56	(2%)	**113**	**(2%)**
Netherlands	12	(2%)	11	(1%)	20	(1%)	52	(2%)	**95**	**(2%)**
Italy	1	(0%)	7	(1%)	6	(0%)	76	(3%)	**90**	**(1%)**
Turkey	1	(0%)	3	(0%)	41	(3%)	33	(1%)	**78**	**(1%)**
Other	42	(6%)	88	(7%)	167	(10%)	512	(19%)	**809**	**(13%)**
Unknown	180	(25%)	284	(23%)	48	(3%)	38	(1%)	**550**	**(9%)**
Total*	**731**		**1225**		**1604**		**2648**		**6208**	

*2017 data are extrapolated based on the first eight months of the year. Without extrapolation, the total number of publications is 6,110.

**Fig 5:**
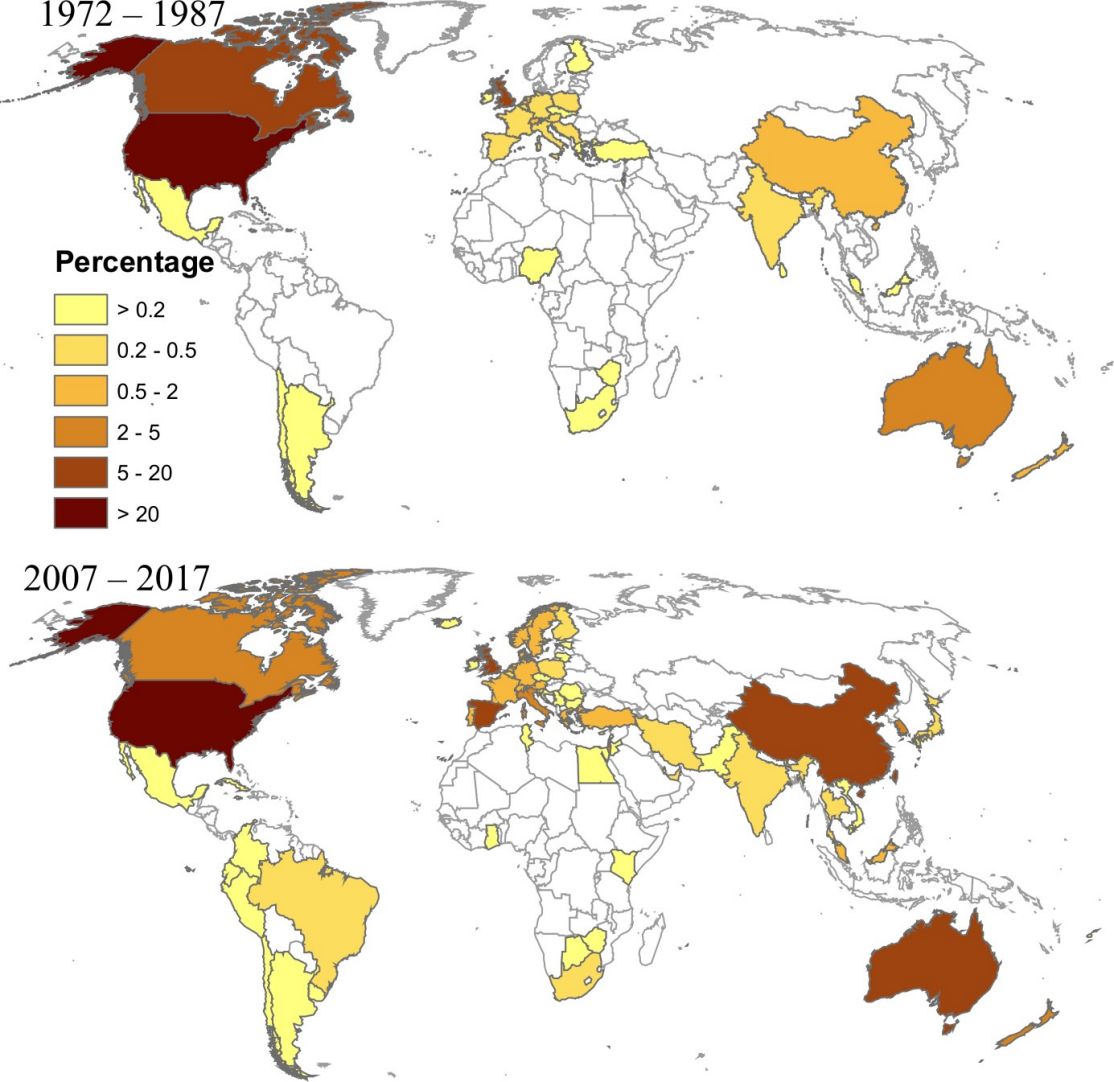
Temporal change in geography of tourism research published in *ATR*, *JTR*, and *TM* by first author’s affiliation. The color scale indicates percentage of papers coming from a specific country; papers with unknown authors’ affiliation are not taken into account. Mainland China, Taiwan, Hong Kong, and Macao are presented separately, following Scopus database format.

The distribution pattern in Fig 4 and the numbers from Table 4 indicate that articles from the US, the UK, Australia, and Canada account for a large portion of tourism scholarship, especially in the beginning of the study period. However, their relative combined output dropped from 64% in 1978–1987 to 43% in 2008–2017, while scholarship from countries such as Mainland China, Spain, and Taiwan grew noticeably. In particular, Mainland China increased its output from 1% in 1978–1987 to 5% in 2008–2017, or even more if we look only at the last few years depicted in Fig 4. It is also worth noting that the Other category (Table 4) accounts for 19% of the total scholarly output in the last period, compared to 6% at the beginning. The Other category includes 70 countries, of which the ten largest contributors are Austria, Norway, France, Portugal, Sweden, Germany, Macao, Switzerland, Singapore, and Greece. The authors also visualized dynamics of geographical representation of various world regions (by the location of the first author’s institution) in tourism studies, using, for contrast, the earliest (1972–1987) and latest (2007–2017) periods (Fig 5). The expansion to Asian, Middle East, African, and South American regions is clearly visible.

### 4.3 Gender: Who is publishing?

The authors’ gender was identified from their first names using the Genderize.io software, which predicts the probability of a specific name to belong to a certain gender from statistics extracted from social network accounts. For example, Genderize.io has 763 people whose first name is ‘Kim’ in its database; of these, 687 are females. Hence, the estimated probability of a person with the first name ‘Kim’ being a female is 90%. Only the authors whose gender was identified with at least 0.6 probability were retained; the rest of the authors were excluded, which resulted in 5,591 unique authors, including 3,064 unique first authors (which constitutes 79% / 80%, respectively, of the authors/first authors identified through Microsoft Academic search). The authors whose gender could not be clearly identified were mainly (1) those where the Microsoft Academic database included only initials, and (2) those with names from China. We speculate that the latter bias relates to the way the Genderize.io database was built: the gender was extracted from social network registration records. Because Chinese nationals are restricted in their use of the major international social networks such as Twitter and Facebook by the legislative and technological actions, the Genderize.io Chinese name base might be limited.

**Fig 6:**
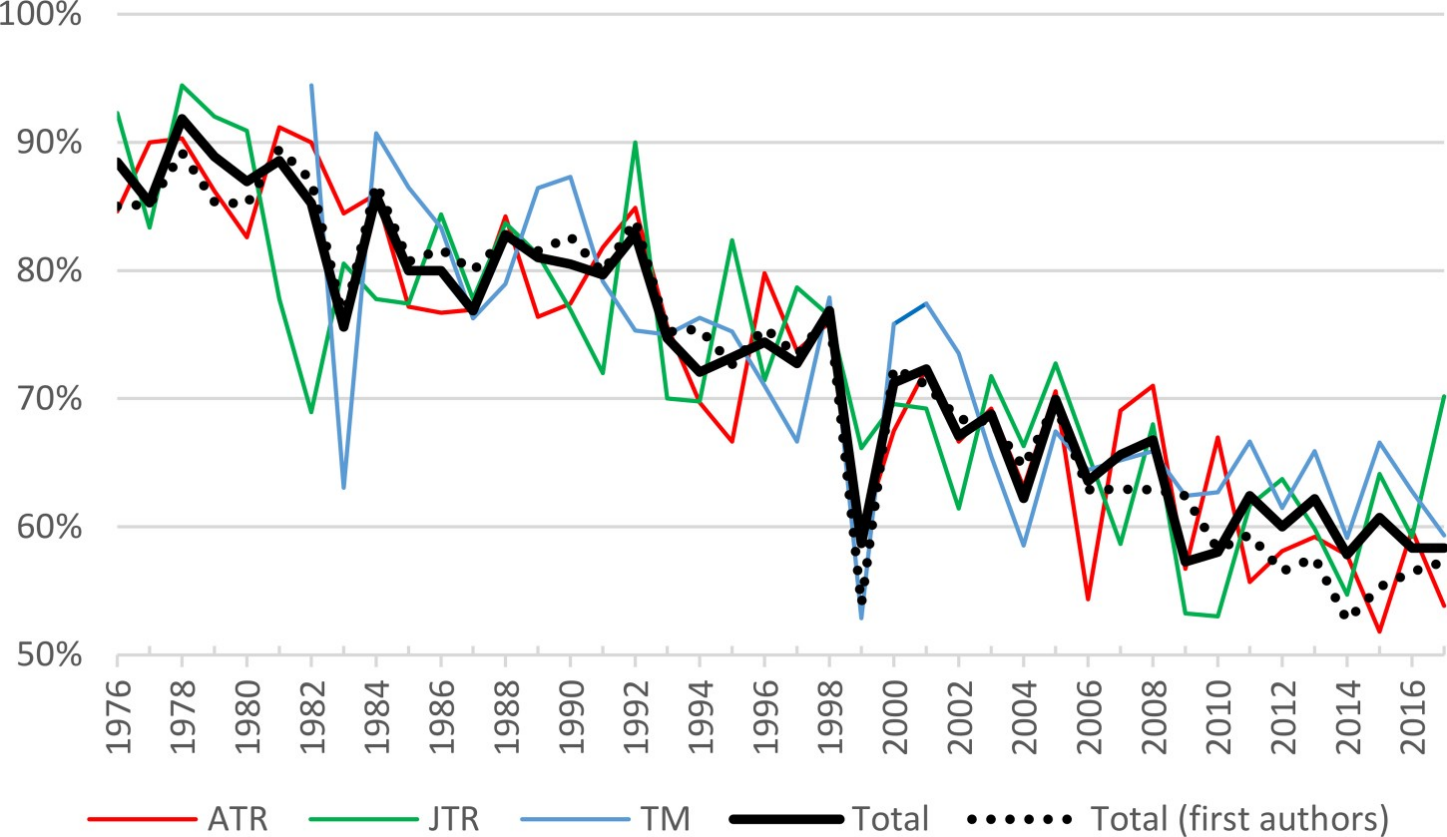
Percentage of male authors in tourism academic publications over time.

The results indicate that the percentage of papers authored by male tourism researchers has fallen steadily throughout the study period, from 93% in the 1970s to 60% in the 2010s (Table 5, column Total). The percentage of male scholars as first authors showed similar dynamics, falling from 93% in the 1970s to 57% in the 2010s (Table 5, column Male First Authors). The corresponding yearly dynamics are shown in Fig 6; note that the figure starts from 1976 to include only those years in which the genders of at least 20 first authors could be identified. Overall, through the years all journals display the same trend of increased share of female tourism scholarship, both within the authorship team and as the first author.

**Table 5:** Gender distribution of authors in tourism scholarship.

	Male Authors, %				Male First Authors %
Years	ATR	JTR	TM	Total	
1970s	88	92	—	93	93
1980s	83	80	82	82	83
1990s	74	75	74	75	75
2000s	66	66	68	66	67
2010s	58	61	63	60	57

## 5. Discussion

Using a text mining approach, namely, Latent Dirichlet Allocation, the study identifies 14 subject areas in tourism scholarship from more than four decades of research. It concludes that LDA is sensitive enough to detect interpretable topics and their trends in large volumes of textual material. Drawing on Mazanec [26], the study contributes to tourism literature by extending methods for identification of subject areas from tracking separate words as indicators of research interest [14,31] to deriving latent topics via text mining algorithms. The findings indicate that the identified subject areas have been in existence for the whole period of analysis, yet fluctuations in interest to those research topics have been detected. It is important to notice that the existence of the same topics during 40 years does not mean that no innovations have occurred in how researchers address pertinent issues of these areas in terms of conceptual foundations, methods of analysis, or geographical context. A more finely granulated analysis would allow identification of more specific and detailed areas of study, as topics can fragment into sub-themes when different parameters for the analysis are chosen.

The most noticeable fluctuations in relative shares of the 14 identified knowledge domains (Fig 4) reflect the growing interest of researchers to the tourist as an individual, rather than tourism industry as a system. This interest transcends in such topics as tourist experience and motivation (topic 8) as well as service quality and satisfaction (topic 5). All three journals, ATR, JTR, and TM, capture this interest to individual tourist experiences, while primarily JTR and TM reflect interest to issues of quality and satisfaction (Table 3). The studies of tourism from the macro perspectives of Tourism industry (topic 4), Modeling and forecasting (topic 6), Market segmentation (topic 9), Governing tourism development (topic 12) demonstrate a steady performance or, as is the case with Tourism demand (topic 11), decreases throughout the years. It is still a question how much the identified dynamics are due to the actual interest of researchers in a particular knowledge domain and how much it is reflective of the “Big Three” journal policies and preferences. One has to bear in mind that four decades ago there were much fewer tourism journals than now; therefore, less “generalistic” and more “specialty” journals like for example *Journal of Sustainable Tourism*, *Tourism Geographies*, or *Tourism Economics* may have drawn towards themselves research on particular topics since they were established. However, since new journals have been created in all the areas of tourism studies in the considered time frame, if is not possible to numerically estimate the influence of their topical distribution to the study findings.

With respect to geographical dynamics of tourism scholarship, the findings indicate that it has been expanding (Figs 4 and 5). In Table 4, the Other category contains a large share of countries, including Macao, Singapore, Malaysia, Japan, South Africa, India, Cyprus, Brazil, Poland, Thailand, and many others that are culturally different from the ‘collective West’ countries of the US, the UK, Australia, Canada, and Western Europe [52]. This expansion does not necessarily mean that a non-Western perspective is expanding as well, since authors from non-Western cultures can adopt a Western worldview, but encouraging nevertheless. Further, the identified themes mostly reflect tourism as an industry, emphasizing marketization and segmentation, management issues, demand and consumption, and hedonic orientation and are largely associated with the Western perspective in the studies of tourism [16]. It also seems that alternative, non-consumption-oriented themes are predominantly reflected in studies classified under the topics ‘tourism as a social phenomenon’ and ‘sustainable tourism.’ To verify this assumption, we examined LDA classifications of three articles presented by Higgins-Desbiolles [16] as reflecting a non-Western perspective on tourism: [53–55].

Inayatullah [55] addresses an Islamic outlook on tourism as *haji*, where “travel or the accumulation of wisdom, *ilm*, is the essence of Islam. Travelling, visiting wise people, finding holy sites, was an integral part of life” (p. 411). The LDA solution classified this paper as sustainable tourism (446), tourism as a social phenomenon (291), tourism experience and motivation (145), and conferences (145) (the weights shown in parentheses total 1,000). Berno [54] studies how Polynesian people from the Cook Islands engaged with tourism and integrated it into their value system. The article was classified as tourism as a social phenomenon (319), tourist experience and motivation (296), sustainable tourism (182), tourism industry (136), and governing tourism development (65). Finally, the article by Allcock and Przeclawski [53] is an introduction to an *ATR* thematic edition on tourism in centrally planned economies; it does not have an abstract and, therefore, was not classified. Consequently, we analyzed another paper [56] by the same first author found in our database. This paper addressed the potential of planned economies for tourism development and was classified as sustainable tourism (234), governing tourism development (214), tourism as a social phenomenon (112), tourism industry (112), tourism demand (88), service quality and satisfaction (65), image and risk (57), tourist experience and motivation (54), market segmentation (30), and conferences (29). The only three topics with zero weights were attractions, modeling and forecasting, and local communities. As evidenced by the provided examples, the assignment into topics is quite solid.

Gender wise, the study has shown that the presence of female scholarship in the body of tourism research has been steadily increasing throughout the years. This finding is consistent across several methods used. Moreover, the citations analysis does not indicate that currently the works by female authors are lesser sited (Table 5). The authors want to note, however, that these results by themselves do not support or refute any claim or statement regarding whether the parity between male and female representation in tourism academia, research, or leadership has been reached [19]. However, the social progress through the years has been clearly demonstrated by the analysis, together with the positive developments in geographic location of research contributors. These two findings, together with identification of dominant subject areas in tourism research in an objective way via text mining methods are considered the main contributions of the article to the tourism literature.

### 5.1. Limitations and future research

It might seem that the solution is highly dependent on number K of topics chosen. However, in the authors’ experience, it is not so. The range of 10–30 solutions was examined, and, while some of the key words were re-distributed across the topics, the main themes were nevertheless present in the solution. It should also be noted that deriving a large number of topics introduces redundancy in interpretation that may be less convenient for making summaries [51]. Yet, using a larger number of topics is possible, as shown by Kirilenko and Stepchenkova [57] who employed principal component analysis to identify themes in public discourse on climate change. Importantly, the LDA approach demonstrated in this paper is scalable. Researchers can select a theme of interest, identify articles that pertain most strongly to this theme, and conduct LDA on that textual corpus to obtain various subtopics of the theme. Furthermore, when researchers know what words they want to track, they can easily do so; for example, it is quite possible to track the dynamics of SEM analysis, sentiment analysis, or some other method in tourism studies.

As can be seen from the examples in the preceding section, some topics have less “face validity” than others, namely, the topic labeled as ‘conferences’. To understand this issue better, note that the authors did not ‘sift through’ the abstracts downloaded from the Scopus database; therefore, not only research articles but other materials such as conference announcements and reports are also present in the database. The decision to keep these materials in the database was based on the following reasons: 1) ‘weeding out’ is largely a manual procedure [5] that is inherently subjective and hence to some degree negates the use of mostly automatic classification; 2) the share of such materials in the total textual corpus is small (our estimation is under six percent); and (3) these materials, while structurally different from research articles, also signal topical interest in tourism as a field of study.

Finally, this research is limited to the "Big Three" tourism journals: the most reputable, highly cited journals with a long history. Because of that, we presumed these journals to be representative of tourism- related literature. However, this might have made our results dependent to some degree on the methodological preferences of these journals’ editorial boards (e.g., for a hypothesis-driven as opposed to a data driven research such as one used as this paper). That raises a possibility of some cutting-edge tourism research topics shifting to other journals, escaping our analysis. One possible example of such topic would be climate change impact on tourism, which is mainly published in Journal of Sustainable Tourism or in high-ranking non-tourism journals such as Climatic Change. A wider data sample would present a clearer picture of the emerging topics and their contribution to overall tourism scholarship.

## Supporting information

S1 File Publication data used in this research. (XLSX)

S2 File Stop words and synonym dictionary. (XLSX)
